# Case Report: Chronic Lymphocytic Leukemia With a Rare Translocation t(14;19)(q32;q13) Involving IGH*/BCL3* Rearrangements: Report of Three Chinese Cases and Literature Review

**DOI:** 10.3389/fonc.2020.594732

**Published:** 2020-11-19

**Authors:** Qinlu Li, Shugang Xing, Heng Zhang, Xia Mao, Min Xiao, Jia Wei, Ying Wang

**Affiliations:** Department of Hematology, Tongji Hospital, Tongji Medical College, Huazhong University of Science and Technology, Wuhan, China

**Keywords:** chronic lymphocytic**** leukemia, complex karyotype, fluorescence *in situ* hybridization, chromosome banding analysis, *BCL3* rearrangement

## Abstract

**Background:**

A translocation t(14;19)(q32;q13) leading to a fusion of IGH and BCL3 which is a rare cytogenetic abnormality in CLL patients, has a more aggressive clinical course with a shorter time to first treatment (TTT) and worse overall survival (OS). To date, there is no literature reporting the identification of the t(14;19) in Chinese CLL patients and the reviewing the characteristic of all patients with this abnormality reported previously in the literature.

**Patients and Methods:**

We first demonstrate three cases of t(14;19) translocation among the 200 CLL patients from 2017 to 2019 in our hospital. We investigated several aspects such as clinicopathologic features, cytogenetic analysis, IGHV mutations, next-generation sequencing technology (NGS), and histopathological characteristics in order to clearly define the features of this entity in Chinese patients and compare them with patients reported previously in western countries.

**Results:**

The clinical and pathological features of our three cases resemble those of earlier reports. All patients had atypical morphologic features and atypical immunophenotypes with low CLL scores detected by flow cytometry. All cases were unmutated in the IGHV mutations. Two cases showed complex karyotype and one case demonstrate missense mutations of TP53 and FBXW7.

**Conclusion:**

In conclusion, this is the first report on IGH/BCL3-positive B-CLLs in Chinese people, which provided a comprehensive analysis of clinical and pathological characteristics. In addition to some similar clinical and laboratory features reported in the previous literature, we first found that CLL with t(14;19) has a higher possibility of being accompanied with high complex karyotype (high-CK), which is now regarded as a novel negative prognostic marker. Early identification of this abnormality in CLL patients is so important that patients can benefit from the more aggressive treatments at the onset of the disease.

## Clinical Practice Points

1. t(14;19)(q32;q13), involving the *BCL3* locus at chromosome 19q13 and the immunoglobulin heavy chain gene at 14q32, is a rare recurrent cytogenetic abnormality identified in chronic lymphocytic leukemia (CLL) cases, most of which are distinctive, classic CLL and are associated with poor prognosis.
2. Our three Chinese CLL patients with t(14;19) also demonstrate atypical morphologic features, immunophenotypes, and progressive disease processes, similar to cases in previous reports from Western countries.
3. It is noteworthy that CLL with t(14;19) is often accompanied with high complex karyotype (high-CK), which is now regarded as a novel negative prognostic marker.
4. Cytogenetic analysis should be performed in all newly diagnosed CLL patients. Furthermore, a probe for *BCL3* for fluorescence *in situ* hybridization (FISH) should be used to increase the detection of t(14;19) in CLL patients, especially when the conventional chromosome banding analysis demonstrates add(14q32) or FISH indicates an abnormal IGH signal.
5. Active treatments, such as Bruton’s tyrosine kinase (BTK) inhibitors, should be considered to block this progressive process once this abnormality is identified in CLL patients.

## Introduction

Chronic lymphocytic leukemia (CLL) is a common clonal neoplasm of small, mature B lymphocytes among adults in Western countries; however, it is relatively rare in Asians (1). Recently, there has been an increasing frequency of new CLL cases in China with the development of the Chinese economy and the improvement of testing methods (2). Cytogenetic analyses, including fluorescence *in situ* hybridization (FISH) and chromosome banding analysis (CBA), play an important role in the prognostic evaluation of CLL. More than 80% of CLL patients harbor at cytogenetic aberrations (3). Among these aberrations, a rare translocation t(14;19)(q32;q13) leading to a fusion of *IGH* and *BCL3*, which was initially reported by Bloomfield et al. in 1983 (4), has been described as the second most frequent translocation involving *IGH* after IgH/*BCL2* in CLL patients, and has a more aggressive clinical course with a shorter time to first treatment and worse overall survival (5–7).

To date, studies reporting the identification of t(14;19) in Chinese CLL patients, use of next-generation sequencing (NGS) for this condition, and application of the novel drug, BTK inhibitor, in patients with this abnormality are lacking. Moreover, previous studies have revealed that Chinese CLL patients display different clinical and molecular features from CLL patients from Western countries (8, 9). We first demonstrate here three cases of t(14; 19) translocation among 200 CLL patients from 2017 to 2019 in our hospital. We performed several investigations, including assessment of clinicopathologic features, cytogenetic analysis, IGHV mutation analysis, NGS, and histopathological review of *IGH*/*BCL3*-positive cases in our department to clearly define the features of this entity in Chinese patients and compare them with those of patients from Western countries.

## Case Description

From June 2017 to December 2019, we received 200 blood and bone marrow samples from patients diagnosed with CLL according to the iwCLL criteria in our laboratory. Using the standard Wright-Giemsa staining protocol, peripheral blood smears were prepared for manual 100-cell differential white blood cell counting; manual 200-cell differential white blood cell counting was performed using bone marrow aspirate smears. Particular attention was given to the features of lymphocytes. Bone marrow core biopsy and excisional lymph node biopsy specimens, when available, were routinely processed, and H&E-stained sections were examined. Samples from all patients underwent conventional G-banding chromosomal analysis by culturing for 72 h with the stimulation of CPG mitogens (the combination of CpG-oligonucleotide DSP30 and interleukin-2). FISH was performed in all patients using a commercially available probe panel (MetaSystems, Altlussheim, Germany), including del(13q14)/D13S19, +12, del(11q22)/ATM, and del(17p13)/*TP53*, t(11;14)(q13;q32)/*IGH*/*CCND1*. Peripheral blood or bone marrow aspirate samples were analyzed by flow cytometry (FACS Calibur), and the CLL score was calculated according to the system by Matutes and colleagues (10). Scores were based on five variables: dim expression of surface immunoglobulin, 1 point; CD5, 1 point; CD23, 1 point; dim or absent CD22/CD79b, 1 point; and absent FMC-7, 1 point. Total RNA from bone marrow aspirate or peripheral blood was extracted for sequence analysis of IGHV genes using the International ImMunoGeneTics (IMGT) information system and database tools (IMGT/V-Quest, http://imgt.org). The IGHV mutation statuses were designated as follows: unmutated if there were <2% mutations (≥98% homology) or mutated if there were >2% mutations (≤98% homology) compared to germline sequences. NGS was performed using Ion Torren PGM/Illumina NextSeq 550Dx platform with the targeted panel from 40 genes (*ATM, BIRC3, BTK, CALR, CARD11, CD79A, CD79B, CHD2, CSMD3, CXCR4, DDX3X, EZH2, FAT1, FBXW7, KLHL6, LRP1B, MAPK1, MUC2, MYD88, NOTCH1, PLCG2, PLEKHG5, POT1, SF3B1, SPEN, TGM7, TP53, XPO1*, and *ZMYM3*). Finally, we retrospectively screened and identified three cases with t(14; 19) in the cytogenetic database. IGH and *BCL3* gene rearrangement probes (Metasystems, Altlussheim, Germany) were used to prove that all the cases had IGH/*BCL3* translocation. Clinical information and related laboratory testing data were collected together and are described in **Table 1** and **Table 2**, respectively.

**Table 1:** Clinical features.

Case No.	Age (years)	Sex	LymphocyteCount (×10^9^)	Hb(g/l)	PlateletCount (×10 ^9^)	Rai Stage	LN	Spleen	Therapy	Outcome(survivalin months)
1	50	M	15.98	123	310	0	No	No	Observation for 18 months FCR 5 cycles	Alive (26)
2	46	M	16.85	105	258	III	Yes	No	FCR 4 cycles	Alive (17)
3	55	M	65.29	67	95	IV	Yes	No	FCR 2 cycles BTK inhibitor orelabrutinib	Dead (32)

LN, lymph nodes; FCR, fludarabine, cyclophosphamide, rituximab; M, male; Hb, hemoglobin.

**Table 2:** Laboratory results.

Case		BM Cytology	Lymph Node Results	Flow Score	IGHV Mutation Status	NGS (VAF)	Karyotype	FISH	
								IGH/BCL3(% cells positive)	CLL panel(% cells positive)
1		Heterogeneous mixture of small and medium cells with indented nuclei	ND	3	U	ND	47,XY,+12,t(14;19)(q32;q13)[10]	Pos (45%)	+12 (50%)
2		Atypical; increased large cells with irregular nuclear	SLL with increased large cells	3	U	ND	85~90, XXYY, del(6)(q21),-7,-8,-10, +12,-13,t(14;19)(q32;q13)×2,-15,-18[cp10]	Pos (60%)	+12; (62%) del (13q) (68%)
3		Atypical; abundant cytoplasm, nuclear indentation	ND	3~4	U	TP53, c.733G>A, p. Gly245Ser (p. G245S);(3.4%) c.713G>A, p. Cys238Tyr (p.C238Y)(55.8%) FBXW7,c.1393C>T, p Arg465Cys (p.R465C)(47.1%)	44,XY,t(6;8)(q13;p21),t(14;19)(q32;q13), add(17)(p13),-18,-20[2]/46, XY[18]	Pos (35%)	del(13q)(40%) del TP53 (38%)

BM, bone marrow; CLL, chronic lymphocytic leukemia; SLL, small lymphocytic leukemia; U, unmutated; ND, not done; FISH, fluorescence in situ hybridization; Pos, positive; VAF, variant allele fraction.

### Patient 1

Patient 1 was a 50-year-old man admitted to the hospital for hemorrhoidectomy in December 2017. The blood examination showed high white blood count, 30.75×10^9^ cells/L. The other parameters were absolute lymphocyte count, 25.95×10^9^ cells/L; hemoglobin level, 101 g/L; and platelet count, 241×10^9^ cells/L. The patient had no fever, cough, chest tightness, or other discomforts. No enlargement of lymph nodes or the spleen was found in the CT scan. Bone marrow aspirate showed extensive infiltration by a heterogeneous mixture of small and medium cells with indented nuclei. Leukemic cells expressed CD19, CD22, CD23, and kappa, whereas the expression of CD5 and surface Ig was dim-positive and that of FMC7 was negative; the CLL score was 3. By karyotyping, trisomy 12 was the only additional cytogenetic abnormality, and this was confirmed by FISH using a probe panel. Interphase FISH analysis using the dual-color *BCL3* probe demonstrated one intact red/green fusion signal and 1 red and 1 green split signal, which indicated a *BCL3* gene break-apart. The IGHV status was unmutated. The patient was placed in the Rai 0 category. The patient was observed for 18 months without any treatment until the lymphocyte count reached 55.95×10^9^ cells/L. To date, the patient has received five cycles of the standard rituximab, fludarabine, and cyclophosphamide (FCR) treatment and the follow-up is being continued.

### Patient 2

**Figure 1:**
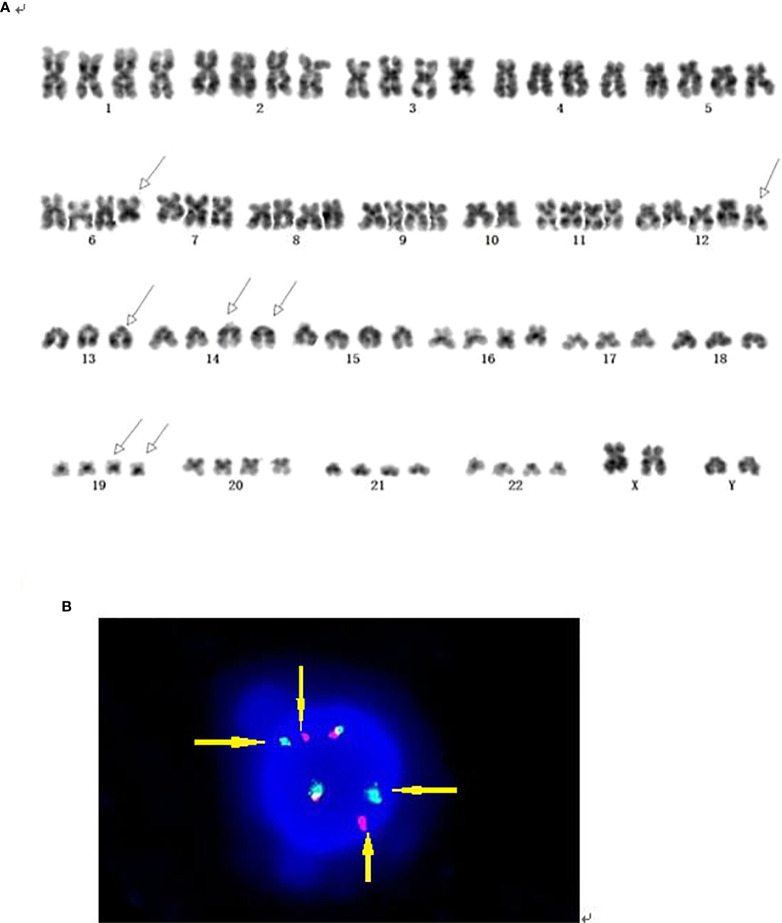
**(A)** G-banding analysis of case 2 showed t(14;19) accompanied by a complex.karyotype of near-tetraploid with concurrent 6q deletion and +12. **(B)** Interphase FISH analysis of case 2 using the dual-color BCL3 probe. FISH results demonstrated two intact red/green fusion signals, and 2 red and 2 green split signals, which indicated BCL3 gene break-apart. (Yellow arrows pointed to split signals of the rearranged gene).

Patient 2 was a 46-year-old man who presented with cervical lymph node enlargement in September 2018. The blood examination showed high white blood count, 20.55×10^9^ cells/L. The other parameters were absolute lymphocyte count, 16.85×10^9^ cells/L; hemoglobin level, 105 g/L; and platelet count, 258×10^9^ cells/L. PET/CT showed multiple lymph node enlargements throughout the body. Lymph node biopsy demonstrated that the lymph node specimen was predominantly composed of small lymphocytes with a mixture of larger lymphocytes with scanty cytoplasm, clumped chromatin, and round or slightly irregular nuclei. These slightly large cells were diffused with an increased proliferation index. Immunohistochemical staining showed that the lymphoid cells were positive for CD5, CD20, CD23, and BCL2 and negative for CD3, CD10, BCL6, and Cyclin D1. The index of Ki-67 staining was about 30%. Bone marrow morphology displayed abnormal lymphocyte proliferation, diffuse or interstitial distribution, small cells mixed with increased large cells, round or irregular nuclei, dispersed chromatin, and insignificant nucleoli. Immunohistochemical staining results were as follows: CD20+, CD5-, CD23 (partial +), CD10-, CD3-, MPO-, BCL2-, and CyclinD1-. G-banding analysis showed a complex karyotype of near-tetraploid: 85~90, XXYY, del(6)(q21), -7, -8, -10, +12, -13, t(14;19)(q32;q13)×2, -15, -18[cp10] (**Figure 1A**), and a concurrent 6q deletion and trisomy 12 in addition to t(14;19), which were all confirmed by FISH (**Figure 1B**). The CLL immunophenotypic score was 3 with positive CD5 and CD23 expression, brighter CD22/CD79b, and brighter surface immunoglobulin expression and negative FMC7 expression. The IGHV status was unmutated. This patient was finally classified into the Rai III category. Due to the lack of funds, the patient was treated with the FCR regimen for four cycles. At the same time, he was considered to have entered the clinical trial of BTK inhibitor.

### Patient 3

Patient 3 was a 55-year-old man diagnosed with CLL at a local hospital and began treatment with chlorambucil without the related prognostic evaluation including CBA, FISH, IgHV status, and NGS in June 2016. One and a half years later, the patient presented to our hospital because of disease progression. After admission, routine blood examination showed a leukocyte count of 80.55×10^9^ cells/L, absolute lymphocyte count 65.29×10^9^ cells/L, hemoglobin level 67 g/L, and platelet count 95 × 10^9^ cells/L. The patient had no superficial lymphadenopathy or splenomegaly. Computed tomography demonstrated bilateral hilar and mediastinal lymph node enlargement. Lymphocytes in the peripheral blood and bone marrow were cytologically atypical, and the majority of cells were small with admixed medium and large cells in a lower nuclear/cytoplasmic ratio and nuclear indentations. G-banding chromosome analysis showed a complex karyotype, 44, XY, t(6;8)(q13;p21), t(14;19)(q32;q13), add(17)(p13), -18, -20[2]/46,XY[18]. FISH further confirmed the existence of the deletion of the P53 gene. Flow cytometry showed 66.87% monoclonal B lymphocytes with the expression of CD5 and CD20, and dim Sig expression as well as negative expression of FMC7, CD79b/CD22, and CD103. The CLL score was 3–4. NGS demonstrated the missense mutations of the seventh exon of *TP53* and the ninth exon of *FBXW7*. The IGHV status was also unmutated. The patient was placed in the Rai IV category. The standard FCR treatment was performed for 2 cycles. Due to the unsatisfactory efficacy of chemotherapy, the patient entered the clinical trial of BTK inhibitor. However, the patient died of serious pulmonary infection after two months of treatment with BTK inhibitor.

## Discussion

t(14;19) (q32;q13) involving *IGH*/*BCL3* gene rearrangements is considered to be a rare and recurrent chromosomal abnormality in B-cell malignancies, especially in CLL patients (6). Until now, only a few small series of reports on this genetic abnormality have been published. These reports suggested that t(14;19)-positive CLL might represent a distinct entity, which exhibits features that are different from those of typical CLL, including younger patient age, an aggressive clinical course, atypical morphologic and immunophenotypic features, and an association with trisomy 12 (6, 7).

To our knowledge, our report is the ﬁrst report of Chinese CLL patients with t(14;19) (q32;q13). The clinical and pathological features of our three cases resemble those of earlier reports. All three patients were very young, with an average age of 50 years, and had atypical cellular morphology with cleaved nuclei or lymphoplasmacytic features. The growth pattern in the bone marrow biopsy specimens was interstitial to diffuse. Flow cytometric immunophenotyping showed an atypical immunophenotype with low CLL scores. All our cases were unmutated based on the molecular genetic analysis of the IGHV somatic mutation status.

Similar to previous studies, all of our cases harbored additional cytogenetic abnormalities in addition to t(14;19), and two of the three cases coexisted with +12 (case 1 and case 2). Moreover, both cases 2 and 3 demonstrated high complex karyotype (high-CK, defined as ≥ 5 abnormalities). Combined with the 86 previously reported CLL cases with t(14;19) (q32;q13) in the literature, we found that 79 cases had complete karyotype information, and high-CK was observed in 20/79 (25.3%) of the cases (**Table 3**), which is remarkably higher than the 271/5290 (5.1%) for common CLL reported in a recent European multicenter study conducted by Panagiotis Baliakas et al., the largest to date (P <0.001) (14). Similarly, trisomy 12 was also found in 39/79 (49.4%) of the cases, which is significantly higher than the 15% reported in the literature for unselected B-CLLs (14). Additionally, trisomy 12 was associated with atypical morphology, atypical immunophenotype (15).

**Table 3:** Clinical and laboratory data of all cases with t(14;19) accompanied by high-CK in previous studies.

Case(reference)	Age/sex	Diagnosis	WBC(lymphcyte×10^9^)	Flow cytometry score	Karyotype	FISH involvingBCL3rearrangement	CLLFISHPanel
1 (11)	53/M	CLL with subsequent PLL	61.9	NA	45,XY,t(6);?(?p25);?,-9,+der(12)t(12;17)(q21.2;q11),-14, +der(14)t(14;19)(q32;q13.1),-17, t(19);?(q13);?/46,XY	NA	+12
2 (11)	67/M	Large cell CLL with immunoblastic features	19(13.11)	NA	47,XY,del(2)(q13q31),-6,+der(6)t(6;9) (q23;q11),-9,+der(9) t(6;9)(q25;q11),del(10)(q22q24),+r(12),t(14;19)(q32;?q13.1)	NA	+12
3 (11)	55/M	Prolymphocytoid transformation of CLL	189(162.54)	NA	45,XY,t(2;14)(p13;q32),-14,+der(14)t(14;19)(q32;q13),-17,-19, +der(19)t(?17;19)(?q21;q13)/46,XY	+SB	+12
4 (11)	45/F	CLL transforming later to high-grade lymphoma after 2 y	74.8	CD19+ CD5+	47,XY,+12,t(14;19)/46,XX; Additional abnormalities with Transformation: add(10)(q24),der(11) t(1;11)(q12;q25),t(12;22)(p13;q11)	NA	NA
5 (11)	51/M	CLL, progressive	11.75	CD20: 43% CD5: 55% FMC7:57%	45,XY,-6,t(14;19)(q32;q13),+der(16) t(16;17)(p11;q11), -17,der(21)t(6;21)(q13;p13)/46,XY	+SB	NA
6 (11)	91/F	CLL transforming to DLBCL	NA	CD20+ CD5+ CD11c+ FMC7 dim+ κ+	47,X,dup(X)(q21q22),del(2)(p23), -4,del(6)(q21q24),+7,add(9)(q34), add(11)(p14),del(13)(q21q31),t(14;19)(q32;q13),add(18)(q32),+mar	+SB	NA
7 (11)	NA	CLL	NA	CD20+ CD5+	47,XX,+12,t(14;19) (q32;q13)/47,idem, add(13)(q34)/45,idem,-5,-18[cp5]	NA	NA
8 (12)	27/F	B-CLL nos (atypical)	NA	Igk+, CD5 ND CD23 ND	46,XX,add(1)(q21),del(6)(q16q22),add(8)(q24),add(10)(p12),t(14;19)(q32;q13.1)[4]/46,XX[4]	IGH/BCL3	NA
9 (12)	61/M	B-CLL nos (atypical)	NA	CD20+, CD23-, Cyclin D1-, CD5 ND	45,XY,der(14)t(14;17)(p13;q12)t(14;19)(q32;q13),der(15)t(3;15)(q21;q26), -17,der(19)t(14;19)(q32;q13)[2]/45,XY,idem,add(15)(p13)[2]/46,XY[2]	IGH/BCL	NA
10 (12)	69/M	B-CLL/SLL-t	NA	CD19+, CD20+, CD5+, CD23+, CD43+, CD38-, CD10-, Igλ weak	44,t(X;6)(p22.2;p21),Y,t(2;22)(p25;q11) del(2)(q34),dic(3;17)(p13;p11),der(6)t(6;14) (q24;q23),add(12)(p13),-14,t(14;19)(q32;q13.1)[11]/88,idemx2[4]/46,XY,add(3)(q28),dup(5)(q15q23), t(14;19)(q32;q13.1),dic(15;17)(p11;p11),+mar[5]/45,XY,dic(4;17)(p11;p11),add(7)(p14),t(14;19)(q32;q13.1)[2]/45,XY,del(2)(p24),add(7)(q36), t(14;19)(q32;q13.1),-17[2]	IGH/BCL	NA
11 (12)	60/F	B-CLL/SLL-t	NA	CD20+, CD5-, CD23-, CD101, Bcl2+	46,XX,dup(1)(q12q31.1),dup(2)(q22q31.3),del(6)(q14q25B26),del(7)(q32q34),del(9)(p12),del(13)(q13q21),t(14;19)(q32;q13),add(14)(q32)	IGH/BCL	NA
12 (12)	72/M	B-CLL nos	NA	NA	46,XY,t(14;19)(q32;q13)[2]/47,idem,+12[3]/47,idem,del(5)(q32)[5]/47,XY,t(2;5)(p24;p14) t(14;19)(q32;q13),+12[3]/46,XY[2]	IGH/BCL	NA
13 (12)	47/M	B-CLL nos	NA	CD19+, CD20+, CD79b+(weak), CD5+, FMC7+, CD25+, CD38+(96%), IgA-,IgD-, IgG-,IgM-, Igλ+,CD23 ND	47,XY,+12,t(14;19)(q32;q13)[4]/46,idem,-Y[2]/46,idem,-Y,add(12)(q?24)[3]/46,idem,-Y, t(12;19)(p?13);?,del(13)(q14;q22)[3]/46,XY[6]	IGH/BCL	NA
14 (5)	53/M	CLL	NA		46,XY,+X,del(X)(q25),add(3)(p21),del(6)(p21),der(8)t(8;17)(p21;q21),-9,-13,t(14;19)(q32;q13.2), +16,-17,+mar[cp19]/46,XY[1]	IGH	del(13q), delP53
15 (5)	74/F	CLL	NA		47~49,XX,+1,der(1)t(1;7)(p22;p13),-2,del(3)(q21),add(4)(q33),+5,del(6)(q21),add(8)(p11.2),add(9) (p23),del(9)(p13),t(14;19)(q32;q13.2),+der(14)t(14;19),del(17)(q23),+1~4mar[cp11]/46,XX[9]	IGH	del(13q),
16 (6)	33/F	CLL/SLL	59	3	47,XX,+12,t(14;19)(q32;q13)[7]/47,XX,+12,add(13)(q34),t(14;19)(q32;q13)[6]/47,XX, del(5)(q33),–5,+12,add(13)(q34),t(14;19)(q32;q13),–18[cp5]/46,XX[2]	IGH/BCL3	NA
17 (6)	40/M	CLL/SLL	11	2	46,XY,t(14;19)(q32;q13.3)[7]/46,XY,t(2;10)(p23;q22),t(14;19)(q32;q13)[3]/46,XY,t(2;10)(p23;q22),del(6)(p21),t(9;20)(q13;p11),t(14;19)(q31;q13)[1]/46,XY[9]	IGH/BCL3	NA
18 (6)	79/M	CLL/SLL	60	3	47,XY,-2,+12,add(14)(q32),t(14;19)(q32;q13),+mar[19]/46,XY[1]	IGH/BCL3	NA
19 (6)	52/M	CLL/SLL	162	3	38-44,XY,del(6)(q21),–8,–13,t(14;19)(q32;q13),–17,–18,+mar[cp13]/46,XY[15]	IGH/BCL3	delP53, -13
20 (13)	59/F	CLL transforming to DLBCL	175.6	NA	44,XX,dup(2)(q21q37),−4,−10,+12,−13,t(14;19)(q32;q13.1),del(17)(p13)[23]/46,XX[2]	IGH/BCL3	+12, delP53, -13

+,positive; -, negative; CLL, chronic lymphocytic leukemia; DLBCL, diffuse large B-cell lymphoma; F, female; FISH, fluorescence in situ hybridization; M, male; NA, not available; NHL, non-Hodgkin lymphoma; ND, not done; SB, cases demonstrating BCL3 rearrangement positivity using Southern blot; SLL, small lymphocytic lymphoma; WBC, white blood cell.

With the introduction of effective mitogens, sufficient metaphases of the CLL clone can be obtained, and thus the detection rate of chromosomal abnormalities has also increased. Cytogenetic abnormalities, particularly high-CK, have recently emerged as one of the independent novel biomarkers associated with an inferior outcome (16, 17). The relationship between CLL patients with t(14;19) (q32;q13) and high-CK remains to be elucidated in larger datasets.

The application of NGS in CLL with t(14; 19) has not been addressed in previous studies. Interestingly, NGS was performed for case 3, and it identified a *TP53* mutation and a *FBXW7* mutation. The *TP53* mutation had high concordance with 17p deletion and was an independent negative prognosis predictor of CLL (18). The frequency of *FBXW7* mutation, which was significantly associated with trisomy 12, was too low to obtain reliable statistics about the prognostic significance in CLL (19). With expansion of our sample size, we will analyze and provide a summary of NGS information for these patients in a future study.

It is well-known that CLL has a highly variable clinical course, with some patients progressing rapidly within several months of diagnosis and others living without treatment for years. Therapy for CLL is currently undergoing a revolution with the introduction of B-cell receptor signaling inhibitors (BTK inhibitors), which have been recommended as the first-line treatment for CLL patients and relapsed refractory patients by NCCN guidelines. The molecular identification of t(14;19) was illuminated by McKeithan et al. (15), and this translocation is associated with overexpression of the *BCL3* gene, which encodes an IKB-like protein and modulates the activity of the NF-KB transcription factors. BTK inhibitors play a role in blocking the NF-KB pathway, so this could also be an alternative treatment for this progressive group of patients. Our patient 3 was already in poor condition when he was enrolled in the BTK inhibitor clinical trial. The duration of drug treatment was too short to evaluate its efficacy. Although we are not yet able to draw a conclusion on the efficacy of BTK inhibitors in patients with t (14; 19), we still suggest that this novel drug or an association of BTKi and anti-*BCL2.* is a good attempt for this kind of patients especially in the early time of the disease. Notably, an overall assessment of the patient is necessary at the onset of the disease. Meanwhile, we could consider the more aggressive treatment once the high-risk factor was identified.

Inevitably, as a retrospective analysis, there are still some limitations in our research, including unavailability of some examination information and short follow-up time, both of which could be overcome with the expansion of our sample size and targeted management of these patients.

In conclusion, this is the first report of *IGH*/*BCL3*-positive B-CLLs in Chinese patients, which provided a comprehensive analysis of clinical and pathological characteristics. In addition to having some similar clinical and laboratory features to cases reported in the literature, we first found that CLL with t(14;19) has a higher possibility of being accompanied with high-CK, which is now regarded as a novel predictive marker for refractoriness to not only chemotherapy-based treatment regimens but also novel agents such as the *BCL-2* inhibitor venetoclax, independent of the presence of *TP53* aberration. Further studies on the pathogenesis of t(14;19) in CLL patients and subdivision of this group of patients combined with different karyotypes or NGS results are required to make more accurate assessments of these patients who could benefit from tailored treatment.

## Data Availability Statement

The original contributions presented in the study are publicly available. This data can be found here: https://www.ncbi.nlm.nih.gov/sra/?term=PRJNA669583.

## Ethics Statement

The studies involving human participants were reviewed and approved by Medical Ethics Committee of Tongji Hospital, Tongji Medical College, Huazhong University of Science and Technology. The patients/participants provided their written informed consent to participate in this study. Written informed consent was obtained from the individual(s) for the publication of any potentially identifiable images or data included in this article.

## Author Contributions

QL designed the study and wrote the manuscript. YW reviewed all related literature, performed bioinformatic analysis, and revised the manuscript in this study. SX performed FISH experiment and gathered related data. HZ made chromosome banding analysis. XM performed the flow cytometry test. MX performed high throughput sequencing experiment. JW collected clinical sample and related data. All authors contributed to the article and approved the submitted version.

## Conflict of Interest

The authors declare that the research was conducted in the absence of any commercial or financial relationships that could be construed as a potential conflict of interest.
